# The Determinants of the Human Milk Metabolome and Its Role in Infant Health

**DOI:** 10.3390/metabo10020077

**Published:** 2020-02-20

**Authors:** Anna Ojo-Okunola, Stefano Cacciatore, Mark P. Nicol, Elloise du Toit

**Affiliations:** 1 Division of Medical Microbiology, Department of Pathology, University of Cape Town, Observatory, Cape Town 7925, South Africa; Mark.Nicol@uwa.edu.au (M.P.N.); elloisedutoit@gmail.com (E.d.T.); 2 International Centre for Genetic Engineering and Biotechnology (ICGEB), Observatory, Cape Town 7925, South Africa; Stefano.Cacciatore@icgeb.org; 3 Institute for Reproductive and Developmental Biology, Imperial College London, London SW7 2AZ, UK; 4 Institute of Infectious Disease and Molecular Medicine, Faculty of Health Sciences, University of Cape Town, Observatory, Cape Town 7925, South Africa; 5 School of Biomedical Sciences, Division of Infection and Immunity, The University of Western Australia, M504, Perth, WA 6009, Australia

**Keywords:** metabolome, human milk, metabolite profiles, human immunodeficiency virus, mastitis

## Abstract

Human milk is needed for optimal growth as it satisfies both the nutritional and biological needs of an infant. The established relationship between breastfeeding and an infant’s health is attributable to the nutritional and non-nutritional, functional components of human milk including metabolites such as the lipids, amino acids, biogenic amines and carbohydrates. These components have diverse roles, including protecting the infant against infections and guiding the development of the infant’s immature immune system. In this review, we provide an in-depth and updated insight into the immune modulatory and anti-infective role of human milk metabolites and their effects on infant health and development. We also review the literature on potential determinants of the human milk metabolome, including maternal infectious diseases such as human immunodeficiency virus and mastitis.

## 1. Introduction

Breastfeeding (BF) emerged as an evolutionary strategy subsequent to the divergence of mammals millions of years ago and natural selection has made milk uniquely suited to nourish infants of each species, with human milk (HM) having constituents different from other mammalian milks [1]. HM is the optimal nutrition for infants, and WHO recommends exclusive breastfeeding (EBF) for the first 6 months of life [2] with continued breastfeeding (BF) up to two years of age. EBF especially during the first 6 months of life, has a protective effect against lower respiratory tract infection (RTI) and mortality due to diarrheal illness in developing countries [3,4]. The health benefits of an exclusive HM diet are maintained even when HM is administered through bottles or other feeding devices. In preterm infants, exclusive HM feeding lessens the likelihood of bronchopulmonary dysplasia [5]. Exclusive HM has also been linked to a reduction in infant morbidity and mortality in the first year of life in preterm infants or (very) low birth weight infants [6,7]. 

The benefits of BF an infant are long-lasting, and extend beyond the period of BF [8]. For example, BF has been shown to be beneficial for cardiovascular health and for the prevention of hypertension and type I and II diabetes during childhood and later adolescent life [9]. Also, EBF has been linked to a lower risk of childhood obesity, which is fast becoming a global epidemic [10]. It has also been estimated that by increasing the rate of BF in low and middle-income countries (LMIC), 823,000 child deaths per year due to gastrointestinal disorders could be prevented [11]. 

In full term infants, HM is almost always sufficient to provide essential nutrients for infant growth and development, irrespective of the mother’s own nutritional status [12]. The components of HM including water, microbes, immunological factors and the metabolome all play important roles in the beneficial properties of BF. 

## 2. Human Milk Metabolome

HM metabolome is defined as the complete complement of all low molecular weight molecule (<1500 Da) within the HM niche. These may include the intermediate and end products of metabolism, which originate from different metabolic processes in the mammary gland [13]. Some metabolites, such as lactose, are produced in the endoplasmic reticulum of the milk secretory cells [14]. Other metabolites are produced through the metabolic processes of resident microbes in HM or originate from other cell types and are filtered through the mammary epithelium from the bloodstream [15,16]. While the abundance of some metabolites, such as fatty acids, is highly variable, the abundance of other metabolites, e.g., urea, is highly conserved suggesting a specific functional role in the infant [16]. This review describes the nutritional and non-nutritional biologically active metabolites of HM, their determinants and their role in infant immune development, gut microbial colonization, and infant health and developmental outcomes. 

The HM metabolome can be investigated using two main technical approaches: proton nuclear magnetic resonance spectroscopy (1H NMR) and mass spectrometry (MS). 1H-NMR spectroscopy has the following properties: (1) it is a highly reproducible technique that specifically identifies small molecule in a non-targeted, non-destructive manner from biological fluids; (2) it provides information about the chemical structure of compounds; and (3) compound coverage is skewed towards the detection of hydrophilic and uncharged compounds, such as sugars [17,18]. The main limitation of 1H-NMR spectroscopy is its reduced sensitivity compared to MS-based approaches [19].

MS-based approaches, on the other hand, are (1) highly sensitive, with a detection limit in the nanomolar range; (2) highly versatile due to a range of instrumental configurations; and (3) the recommended technique for metabolite quantification. When coupled with gas chromatography, it is preferred for the detection of volatile (no need for chemical derivatization), heat-stable compounds of less than 500 Da including amino acids, alcohols and organic acids. MS coupled with liquid chromatography, however, detects larger size compounds (800–2000 Da) with a higher accuracy than gas-chromatography approaches, and without need of chemical derivatization. The main limitation of MS-based approaches is their lack of compound annotation/identification and reproducibility in untargeted studies [19,20]. Overall, both techniques can be used in a complementary manner to maximize the identification of different metabolites within the HM sample.

The first metabolomic study on HM was conducted in 2012 by Marincola et al. [21] using both NMR spectroscopy and gas chromatography-mass spectrometry (GC-MS) to study the aqueous and lipid fractions, respectively, of preterm HM. Compared to the metabolites in preterm formula milk, the aqueous extracts of preterm HM were shown to have high lactose concentrations. The preterm formula milk, on the other hand, had a higher concentration of maltose [21,22]. The HM metabolome was further explored by Practico et al. [23] with NMR spectroscopy. Twenty HM samples were analyzed and a total of 43 metabolites were detected at least once in all HM samples including amino acids, short chain fatty acids, oligosaccharides, sugars, phenolic compounds, tricarboxylic acid (TCA) cycle intermediates and N-trimethyl moieties (choline and acetyl-L-carnitine) [23]. 

Using mass spectrometry coupled with both gas chromatography and liquid chromatography, Qian et al. [24] confirmed the unique metabolic profile of HM and highlighted the significant differences between HM and bovine or formula milk in relation to both the presence and abundance of most metabolites [24]. For example, non-esterified fatty acids such as saturated fatty acids (SFA) with aliphatic tails <16 carbons (capric acid, myristic acid, lauric acid), mono-unsaturated fatty acids (oleic acid, palmitoleic acid, eicosenoic acid) and essential polyunsaturated fatty acids (PUFAs) such as linoleic acid and α-linolenic acid were highly abundant in HM compared with bovine or formula milk [24]. 

## 3. Specific Human Milk (HM) Metabolites and Their Role in Health

### 3.1. Carbohydrates

#### 3.1.1. Lactose

Lactose represents the most abundant metabolite and the major carbohydrate in HM [25]. It is the primary source of calories, providing about 40% of total energy value of HM to the infant. It is the main osmotic component regulating HM volume by drawing water into the intracellular secretory vesicles [16,25]. Lactose concentration ranges between 6.7–7.8 g/dL in mature HM, and its concentration is the least variable of the macronutrients as it is tightly regulated by the mammary gland [26,27]. 

Lactose induces natural responses by upregulating the gut’s antimicrobial peptides, as demonstrated in the T84 colonic cell line and in the THP-1 monocyte-like cell line. This induction may promote intestinal homeostasis and protection of the gut against pathogenic micro-organisms [28]. In addition, a recent study has demonstrated an antagonistic effect of lactose on the growth of specific bacteria. The concentration of lactose in HM was shown to be negatively correlated with the HM bacterial genera, *Enterobacter* spp. and *Actinomyces* spp. [29], which contain important opportunistic and multi-resistant bacterial species [30,31]. 

Lactose concentration has been shown to be positively correlated with the volume of HM intake and the number of HM feeds per day [32,33]. Unlike lipids, lactose concentration was shown to be positively associated with infant weight and adiposity gains between 3 and 12 months of life [32]. Also, the influence of HM lactose on infant growth has been confirmed in a statistical model of a newborn baby (from birth to age 6 months) which simulates the baby’s metabolism of HM to understand the mechanisms of infant growth [34]. 

Lactose also forms part of many carbohydrate-based bioactive compounds in HM, such as forming the backbone of oligosaccharides [12]. Lactose aids mineral absorption in infants including calcium, copper, magnesium and manganese [35].

#### 3.1.2. Human Milk Oligosaccharides 

Human milk oligosaccharides (HMOs) are a group of bioactive compounds representing the third most abundant metabolite in HM, after lactose and lipids [36]. Compared to other mammalian milk, HM contains a higher variety (>200), and more complex structures of soluble oligosaccharides, of which 162 chemical structures have been characterized [37]. Although diverse and structurally complex, each lactating mother synthesizes a unique compositional subset [38]. 

**Figure 1:**
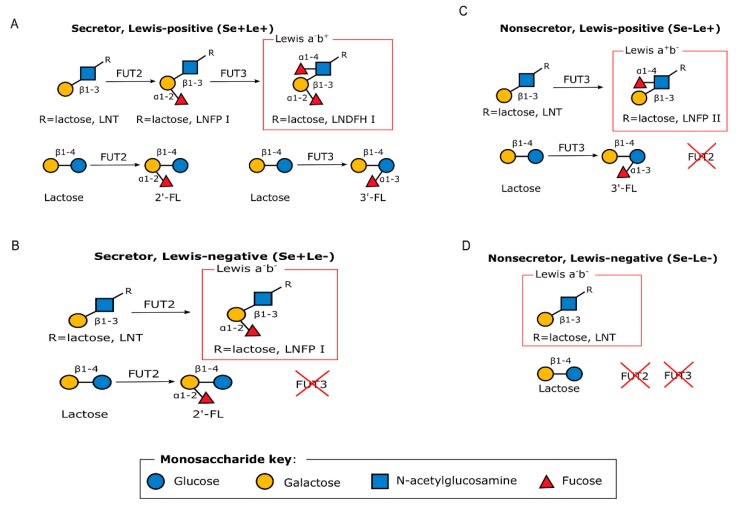
Graphical representation of the enzymatic processes that lead to the different HMO phenotypes. Adapted with permission from Bode 2012 [41]. (**A**) Secretor, Lewis-positive (Se+Le+), (**B**) Secretor, Lewis-negative (Se+Le−), (**C**) Non-secretor, Lewis-positive (Se−Le+), and (**D**) Non-secretor, Lewis-negative (Se−Le−) FUT2: Fucosyltransferase 2; FUT3: Fucosyltransferase 3. 2’-FL: 2’-fucosyllactose; 3’-FL: 3’-fucosyllactose.

HMOs are complex sugars with a lactose core at the reducing end, and are differentiated based on the linkages with one or more building blocks: D-glucose (Glc), L-fucose (Fuc), D-galactose (Gal), N-acetylglucosamine (GlcNAc) or N-acetylneuraminic acid (Neu5Ac) residues [36]. HMO composition depends, in part, upon the expression of two specific genes and as such, individual women synthesize different sets of oligosaccharides [16]. For example, an α 1, 2-linkage attaches fucose residues to a lactose core, in a reaction catalyzed by fucosyltransferase 2 (FUT2) and encoded by the secretor gene (*Se*) or by means of α 1, 3/4-linkages catalyzed by fucosyltransferase 3 (FUT3) and encoded by the Lewis gene (*Le*) (Figure 1) [16]. Women can therefore be differentiated into being a secretor or a non-secretor [39] depending on the expression of active FUT2 enzymes. As such, women can have one of four different phenotypes, namely, Lewis-positive secretors (Se+Le+), Lewis-negative secretors (Se+Le−), Lewis-positive non-secretors (Se−Le+) or Lewis-negative non-secretors (Se−Le−) depending on the expression of FUT2 and/or FUT3 gene as shown in Figure 1 [40]. Non-secretors have low or undetectable concentrations of 2’-fucosyllactose (2’-FL), lactodifucotetraose (LDFT), lacto-N-fucopentaose (LNFP) I, or lacto-N-difucohexaose (LNDFHI) I in their HM, which are all α-1,2-linked fucosylated HMOs [16].

HMOs act as prebiotics, which selectively stimulate the function of beneficial bacteria such as *Bifidobacterium* spp. while suppressing the growth of pathogens [42]. HMOs are resistant to digestion during their passage through the alimentary canal as human infants lack the glycolytic enzymes needed to break them down. Instead, they are digested by certain commensal bacteria in the infant gut to produce short chain fatty acids (SCFAs), which help in establishing a stable ecosystem in an infant’s gut by modulating the immune system and promoting the gut epithelial barrier function [43,44]. SCFAs drive the development and function of regulatory T (Treg) cells, thereby limiting intestinal inflammation. They also serve as an energy source for the epithelial cells of the colon and produce an acidic milieu in the gut, making it inhospitable to potentially pathogenic microbes [45,46]. SCFAs such as butyrate, regulate gene expression through inhibition of histone deacetylase. This action may lead to the inhibition of interferon-γ production and suppression of nuclear factor κB (NF-kB) activation in human colonic epithelial cells [47]. NF-κB, for example, is responsible for early immune inflammatory responses and its dysregulation is seen in inflammatory bowel diseases (IBDs) [48]. 

HMOs inhibit many enteric pathogens, such as toxin-producing *Escherichia coli*, which causes diarrhea in infants [49], *Campylobacter* spp. [50] and *Norovirus* spp. [51] by acting as a decoy receptor for pathogens. HMOs are structurally related to glycans on the intestinal epithelial cell surfaces, to which pathogenic bacteria bind. Breastfed infants, for example, are protected against *Campylobacter jejuni*, which causes diarrhea, as 2’-fucosyllactosamine found on intestinal cells, which acts as a receptor for *C. jejuni*, is also present on HMOs [43,52]. HMOs have also been shown to reduce attachment of the protozoan parasite, *Entamoeba histolytica* (*E. histolytica*), and associated cell toxicity in a human colon adenocarcinoma cell line. The main adhesion-related virulence factor in *E. histolytica* is a lectin that binds with galactose and N-acetyl-galactosamine, and HMOs containing terminal galactose, such as Lacto-N-tetraose (LNT), act as decoy receptors [53]. Cell culture studies have shown that pooled HMOs reduce the invasion and virulence of *Candida albicans* in premature human enteric epithelial cells when *C. albicans* is inoculated onto the epithelial cells, by slowing down the formation of hyphae [54]. 

Different groups of HMOs often exhibit specific properties. For example, sialylated HMOs provide sialic acid needed for neural development, function and cognition [41]. Also, a significantly lower concentration of disialyllacto-N-tetraose (DSLNT) has been observed in HM of mothers whose infants developed necrotizing enterocolitis compared to healthy infants, suggesting a possible protective role of DSLNT to the intestinal tissues of preterm infants and very low birth weight infants [55]. 

### 3.2. Lipids

Lipids in HM are complex and diverse and provide 45%–55% of the total energy needed by infants to support optimal growth [56]. Compared to other macronutrients, the lipid composition of HM is highly variable, and can be affected by duration of breastfeeding, time of day, stage of lactation, maternal nutritional status, and particularly, maternal diet (related to geographic location) [56,57,58]. 

Lipids play a role in membrane structure, signal transmission and cell recognition in signaling pathways, lipoprotein metabolism and as a source and carrier of lipid-soluble vitamins [59]. For example, palmitic acid, which is a saturated fatty acid, is not only an energy source for the infant but also acts as a pulmonary surfactant that reduces the surface tension at the alveolar air–liquid interface and prevents collapse of alveoli [60]. Triacylglycerides are the most abundant lipids in HM. The remaining lipid classes include diacylglycerides, monoacylglycerides, cholesterol, phospholipids (phosphatidylinositol, phosphatidylserine, sphingomyelin, phosphatidylcholine, phosphatidylethanolamine), and free fatty acids (Table 1) [58]. Phospholipids play a vital role in the infant’s immune and inflammatory responses, while sphingomyelin plays a role in central nervous system myelination [61]. In a randomized control trial, low birth weight infants randomized to receive sphingomyelin-fortified milk showed improved neurobehavioral development during infancy compared with the control group, which received only milk [62]. 

All these components make up the milk fat lipid globules (MFLG), which are produced by the alveolar cells of the mammary gland. The MFLG consists of a core of triacylglycerides and a membrane of phospholipids [63]. 

Palmitic acid with a 16-carbon backbone (16:0) is the most common saturated fatty acid in HM and has a preferential positioning of its fatty acids at the sn-2 position, instead of the sn-1,3 positions that are typical of human tissue lipids, infant formula and vegetable oils common in human diets [64]. Studies have shown that this specific position of triglycerides in palmitic acid improves the absorption of both palmitic acid and macro-elements such as calcium and magnesium. This improved absorption decreases constipation and enhances the BF infant’s intestinal well-being [64,65]. 

Lipid fractions in HM include ~34%–47% SFA with principally ~17%–25% of palmitic acid, ~31%–43% monounsaturated fatty acids (MUFA), ~12%–26% n-6 polyunsaturated fatty acids (PUFA), with ~0.8%–3.6% of n-3 PUFA. PUFA are fatty acids containing two or more double bonds along their carbon backbones and include two biologically important subgroups in HM, the n-3 and n-6 essential fatty acids represented by α-linolenic acid (ALA) and linoleic acid (LA), respectively. Both ALA and LA are needed for the growth and maturation of various organs in the infant, especially the brain and eye [66]. Although PUFA have been shown to prevent various allergic diseases in several studies, recent systematic reviews concluded that there is insufficient evidence that HM PUFA influence the risk of childhood allergic and respiratory outcomes [67,68].

ALA can be converted to the long chain-polyunsaturated fatty acids (LC-PUFA), eicosapentaenoic acid (EPA) and docohexaenoic acid (DHA) while LA is converted to arachidonic acid (AA) through consecutive steps involving desaturation and elongation. LC-PUFA of the n-3 and n-6 series are indispensable nutrients with anti-inflammatory and inflammatory activities, respectively [67]. Table 2 below summarizes the major LC-PUFA in HM, their source and functions.

### 3.3. Biogenic Amines 

Biogenic amines are low molecular weight nitrogenous organic bases that are secreted in HM. They include the polyamines (spermine, spermidine and putrescine), together with the monoamines (tyramine) and diamines (histamine and cadaverine) [73]. *Enterococcus*, a major bacteria group in the HM microbiome niche are the main producers of biogenic amines, mainly putrescine and tyramine [74]. The polyamines are the main biogenic amines found in HM, with the most abundant being spermine and spermidine. Polyamines are synthesized from the precursor, ornithine, through the action of the enzyme, ornithine decarboxylase. HM is the first source of exogenous polyamines for the growing infant [75]. Polyamines inhibit cytokine synthesis in human mononuclear cells, downregulating the pro-inflammatory cytokine response [76]. Polyamines are involved in cell growth and proliferation [73,77]. Rapidly growing tissues, such as the mucosa of an infant’s gut require high concentrations of polyamines [75].

HM has a characteristically high concentration of polyamines. However, putrescine and spermidine have been found to be influenced by geographical region [78]. Also, a higher polyamine concentration has been observed in HM of women with preterm babies compared to those with full term babies in early lactation [75], suggesting a greater requirement of this metabolite for the undeveloped intestinal tissues of preterm babies. In a recent study, a positive correlation was observed between putrescine concentrations and *Pseudomonas fragi*, a Gammaproteobacteria in HM [78]. 

A qualitative research and analytical model that assessed the association between HM spermine in the first month of life and allergy appearance estimated that spermine concentrations of at least 5.02 nmol/mL are needed to avert the onset of allergy in breastfed infants [79]. Another study showed that the concentrations of putrescine and spermine were reduced in HM from atopic mothers compared with healthy controls [80].

### 3.4. Nonprotein Nitrogen Molecules

HM nonprotein nitrogen comprises many bioactive molecules including the nucleotides, free amino acids, amino sugars, urea and nucleic acids. These make up approximately 25% of the total nitrogen in HM [61,81,82]. 

#### 3.4.1. Free Amino Acids 

Free amino acids including alanine, glutamate, glutamine, isoleucine, threonine and valine, are more readily absorbed than protein-derived amino acids and they provide a nitrogen source for growing infants [83]. Free amino acids are also known to give HM its unique taste as each free amino acid has its own flavor and can be sensed by receptors in the taste-goblet [24].

Glutamine is the most abundant free amino acid and supplies ketoglutaric acid for the citric acid cycle. It also serves as a major energy substrate for enterocytes and as a brain neurotransmitter [12]. In a recent study, glutamine in HM was positively associated with infant length [84]. Glutamate, on the other hand, is a signaling molecule involved in sustaining gut barrier function and neuroendocrine reflexes. Glutamate serves as an important precursor for other bioactive molecules, including glutathione and proline, and it is also known as an appetite regulator [85]. A previous study has shown a positive correlation between glutamate concentration and maternal pre-pregnancy weight and height [84]. Taurine helps in the structural and functional development of retina receptors and may also aid in fat absorption through its conjugation with bile acids [86]. Taurine is also an organic osmolyte in neural tissues, and it is involved in neural cell volume equilibrium and protection against oxidative stress, thus, preterm infants fed HM have a better neurodevelopmental outcome than infants fed with formula milk due to high taurine content of HM [87]. Together, glutamine, glutamate and taurine make up 50% of the total free amino acids in HM [83].

A recent study showed that most amino acids are influenced by circadian rhythm in mature HM while in colostrum, tryptophan is the only amino acid that showed variation with circadian rhythm, and these amino acids may be helpful in the sleep-wake cycle of infants [88].

#### 3.4.2. Creatine

Creatine is an essential metabolite needed for normal neural development [81]. Neurological symptoms such as speech delay, intellectual disability and epilepsy are displayed in children with inborn errors of creatine synthesis or transport. Creatine helps to buffer cellular adenosine triphosphate (ATP) levels when the ATP synthesis rate is temporarily lower than energy demand [89]. Creatine also transports energy between ATP synthesis sites and where it is being used. Creatine is metabolized to creatinine and excreted in urine. Hence, creatine should be continuously replaced to meet the growing infant’s expanding tissue mass [89]. 

Infants receive their dietary creatine from mother’s HM. Edison et al. [81], however, showed that a breast-fed infant only receives about 9% of the creatine needed from the diet [81]. The remainder is provided through de novo synthesis in the infant whereby creatine is produced from the amino acids, glycine and arginine.

## 4. Determinants of the Human Milk Metabolome

Several maternal and infant-related factors have been identified as potential determinants of the HM metabolome (Table 3). Some of these factors are explored further below. 

(1) Mastitis: Lactational mastitis is a lobular inflammation of the mammary gland caused by *Staphylococcus* spp. and/or *Corynebacterium* spp. with clinical symptoms including fever, chills and localized inflammation of the breast tissue [90]. It often results in early cessation of EBF and may affect up to 33% of lactating women [73]. Mastitis is associated with increased concentrations of the biogenic amines: spermine, putrescine, and histamine [73]. Biogenic amines are usually produced due to the metabolic activity of certain microorganisms including some *Staphylococcus epidermidis* strains with activity for decarboxylation of amino acids; high concentration of biogenic amines in a mastitic breast could be due to increased decarboxylase activity [91]. Mastitis is associated with an increased free fatty acid (FFA) concentration, although no change is observed in the total lipids and phospholipids, suggesting increased lipolysis within HM [92]. The release of FFA in an infected body site is one of the hallmarks of *Staphylococcus aureus* infection; elevated FFA may be a nonspecific immune response to bacteria-associated lactational mastitis [92,93].

(2) Human Immunodeficiency Virus (HIV): Normal HMO composition in HM is disrupted in maternal HIV infection. Several studies show an increased abundance of 3’-sialyllactose (3’-SL) in HM of HIV-positive mothers compared to HIV-negative mothers [55,94,95]. Also, a higher proportion of 3’-SL amongst total HMOs was observed in HM of HIV-negative women with lower CD4 counts [96]. On the other hand, LNT and LNnT were more abundant in HM of HIV-negative mothers [94]. 

Among secretors, significantly higher absolute concentrations of 2’-FL, LNT and LNFP I and higher relative abundance of difucosyllacto-N-tetraose (DFLNT) and fucosyl-disialyllacto-N-hexaose (FDSLNH) was observed in HM of HIV-infected women compared to uninfected secretor women [96]. In non-secretors, only the relative abundance of DFLNH was higher in HM of HIV-infected women compared with HIV-uninfected non-secretors [96].

A higher proportion of 3’-SL per total HMOs in HM has been associated with an increased risk of HIV transmission to infants and with markers of advanced HIV disease, while higher total HMO, LNnT and non-3’-SL HMO concentrations in HM were associated with reduced vertical HIV transmission from mother to child [95]. HMO composition of HM has been found to influence the survival of HIV-exposed, uninfected (HEU) children born to HIV-infected mothers in Zambia up to two years of age [96]. During the BF period, higher concentrations of 2’-FL, 3’-FL, and LNFP I, II/III were significantly associated with reduced mortality among HEU children [96].

(3) Maternal weight: Compared to HM of women with normal weight, HM of overweight mothers had a higher abundance of saturated fatty acid, lower abundance of n-3 PUFA and also, a lower ratio of unsaturated to saturated fatty acids [97]. Lower total polyamines have also been observed in HM of obese mothers compared to HM of mothers with normal body weight [77]. 

(4) Chemotherapy: A case report has shown that DHA and inositol concentrations are reduced in HM of a mother who underwent a chemotherapy cocktail for Hodgkin’s lymphoma as compared with healthy controls [98].

(5) Maternal diet: Dietary intake in the United States is skewed towards omega-6 fatty acids and associated with high content of LA in HM of American mothers, and a particularly low DHA concentration [66]. Unlike an omnivorous diet, a vegetarian diet provides high concentrations of LA and ALA; hence a high concentration of these essential fatty acids in HM of vegans [99]. 

(6) Maternal education: In a study conducted by Nayak et al. (2017) among low income families in an urban area in Bangladesh and India, a higher level of maternal education was associated with decreased saturated fatty acids, and increased ratio of polyunsaturated fatty acids to saturated fatty acids in HM [100], possibly due to better informed food choices.

(7) Geographical location: Significant differences in metabolite profiles have been observed based on geographical locations [78,101,102,103]. With respect to HMOs, HM of Chinese women had highest concentrations of 3’-FL and LNFP III, Spanish women had the highest concentration of 2’-FL, while the highest concentration of LNFP I was observed in HM of women from Finland [101]. Other metabolites including fatty acids, ethanolamine, n-6 PUFA, creatine, lactose and 2-oxoglutarate, pyruvate and lactate, methanol, polyamines, tyrosine have also been found to differ significantly between different geographical locations [78,101,103]. 

(8) Ethnicity: Compared to Caucasian women, black women had higher HM concentrations of 2-oxoglutarate, betaine and glycerophosphocholine and lower HM valine concentration [103]. HM of Caucasians, on the other hand, had significantly lower concentrations of lactate and fucose compared to Asian women [103].

(9) Gestational age: The HM metabolite profile of full term babies shows higher concentrations of carnitine, caprylate, caprate, pantothenate, β-hydroxybutyrate and urea as compared with preterm HM, which has higher concentrations of lactose, phosphocholine, choline, glutamate, DHA, total polyamine, 3’-SL, and 6’-sialylactose (6’-SL) [75,104,105]. 

(10) Stage of lactation: The composition and concentration of HMOs in HM varies over the period of lactation [106,107] with the mean total HMO concentration decreasing from 20.9 g/L in colostrum to 12.9 g/L in mature HM at 4 months postpartum [108]. The mean lactose concentration increases with period of lactation, from 56 g/L on day 4 to 68.9 g/L at 4 months postpartum [108]. 

(11) Course of lactation: The last milk of a feed, the hind HM, has been found to contain up to two or three times the total lipid concentration of foremilk (at the beginning of a feed) [27]. Preterm low birthweight infants have been shown to exhibit increased weight gain when EBF hind HM [109].

(12) Mode of delivery: Though the impact of mode of delivery on metabolite profiles was found to be dependent on geographical location in a recent study [101], overall, women who had a vaginal delivery had significantly higher HM concentrations of 3-hydroxybutyrate and LNFP III, while higher HM concentrations of butyrate, urea, ethanolamine and proline were observed among women who underwent caesarean section (CS) delivery [101]. 

## 5. Conclusions

HM is a rich source of metabolites, which contribute to HM’s beneficial properties and are needed for optimal growth and development of infants. Our current knowledge of the HM metabolome is largely restricted to studies from industrialized/westernized countries, with under-representation of samples from Sub-Saharan Africa. Further studies of geographically and ethnically diverse populations are needed. 

A thorough understanding of HM metabolites, their role in the developing infant and potential determinants is important for our understanding of its nutritional and bioactive value. The application of metabolomics has undoubtedly offered insight into the field of infant nutrition, and its relationship with infant health.

## Figures and Tables

**Table 1:** Lipid classes in human milk (HM). Adapted from [58].

Lipid Classes	Proportion of Total Lipids in HM (%)
Triacylglycerides	98.1–98.8
Phospholipids	0.26–0.8
Cholesterol	0.25–0.34
Non-esterified fatty acids (free fatty acids)	0.08–0.4
Diacylglycerides	0.01–0.7
Monoacylglycerides	Traces

**Table 2:** Long chain-polyunsaturated fatty acids (LC-PUFA) in HM.

Long Chain-Polyunsaturated Fatty Acids (LC-PUFA)	Source, Functions and Determinants	References
*n-3 series*		
Docohexaenoic acid (DHA)	Higher DHA concentration in HM leads to improved neurodevelopmental and vision outcomes in infants. DHA concentration is greatly influenced by dietary intake especially of fish or fish oil supplements in lactating mothers. A dose dependent relationship exists between maternal DHA intake and its concentration in HM, as an increased DHA concentration in HM has been observed in lactating women supplemented with DHA. A lower AA to DHA ratio is found in HM of women from Asian and Scandinavian countries due to higher feeding of DHA-rich fish.	[56,58,66,69,70]
Eicosapentaenoic acid (EPA)	EPA competes with AA for 5-lipoxygenase and cyclooxygenase enzymes needed for the metabolism of AA, thereby antagonizing the pro-inflammatory effects of AA. Lower concentrations of EPA and DHA in HM have been associated with allergy in children.	[71,72]
*n-6 series*		
Arachidonic acid (AA)	AA is the most abundant of the PUFA with ~0.5% of total fatty acids in HM. AA content of HM is relatively stable among women despite variations in diet and lifestyles as it is derived from pre-existing maternal stores. AA serves as a progenitor to signaling molecules, leukotrienes (LTs), thromboxane and the prostaglandins. These products have inflammatory and atherogenic effects on cells. High AA:EPA ratio in HM has been associated with the development of allergy symptoms at 18 months.	[58,71,72]

**Table 3:** Factors that may influence the human milk (HM) metabolome.

Factors Influencing Human Milk (HM) Composition	Metabolome	References
**Maternal Health**		
***HIV***	↑ 3′-SL, ↓ LNT ↓LNnT	[55,94,95]
***Mastitis***	↑ spermine, ↑ putrescine, ↑ histamine, ↑ FFA	[73]
***Pre-eclampsia***	↓ Oligosaccharides, ↓ lactose, ↓ glutamate, ↓ glutamine and ↓ glycerophosphocholine	[110]
***Maternal weight***		
Overweight mothers	↑ SFA, ↓ n-3 PUFA, ↓ Unsaturated FA: Saturated FA	[97]
Obese mothers	↓ Polyamines	[77]
***Medication***		
Chemotherapy	↓ Docosahexaenoic acid, ↓ Inositol	[98]
**Sociodemographic factors**		
***Maternal diet***		
Vegetarian diet	↑ LA, ↑ ALA, ↓ DHA	[66,99]
***Maternal education***		
↑ Higher education	↑ PUFA: SFA	[100]
***Geographical location***		
Chinese women	↑ n-6 PUFA, ↓ SFA, ↑ 3′FL, ↑ LNFP III	[101,102]
Spanish women	↑ 2′-FL, ↑ Putrescine	[78,101]
Finland women	↑ LNFP I, ↑ spermidine	[78,101]
South African women	↑ Lactose, ↑ 2-oxoglutarate, ↑ citrate	[103]
**Infant factors**		
***Gestational age***		
Preterm	↑ DHA, ↑lactose, ↑ HMO conc., ↑phosphocholine, ↑choline, ↑glutamate, ↑3′-SL, ↑ 6′-SL, ↑ polyamine conc., ↓ spermidine/spermine	[75,104,105,111,112,113,114]
Full term	↑carnitine, ↑caprylate, ↑caprate, ↑pantothenate, ↑β-hydroxybutyrate, ↑urea	[104]
**HM factors**		
***Lactational stage***		
Colostrum	↑ HMOs, ↑ LNnT, ↑ 2′-FL, ↑ 3′-SL, ↓ Lactose, ↑ Leucine, ↑ Betaine, and ↑ Creatinine	[43,104,107]
Mature HM	↓ Total HMOs, ↑ 3′-FL, ω6/ ω3 PUFA, ↑ oleic acid, ↑ palmitoleic acid, ↑ linoleic acid, ↑ tri-, di-, mono-glycerides, ↓ cholesterol, ↓ phospholipids, ↓ α-tocopherol, ↓ fucose, ↓ furanose isomers, ↓ D-glucosaminic acid, ↑ Alanine, ↑ caprylate, ↑ caprate, ↑ glutamate	[16,25,100,104,107,115,116]
***Course of lactation***		
Foremilk	↑ free amino acids, ↑ phenylalanine, ↑ threonine, ↑ valine, ↑ alanine, ↑ glutamine, ↑ serine	[109]
***Delivery mode***		
Vaginal delivery	↑ 3-hydroxybutyrate and ↑ LNFP III	[101]
CS delivery	↑ Butyrate, ↑ urea, ↑ putrescine, ↑ ethanolamine and ↑ proline	[101]

↑: Increase concentrations, ↓ Decrease concentrations, SFA: Saturated fatty acids, PUFA: Polyunsaturated fatty acids; FFA: Free fatty acids; LNFP: Lacto-N-fucopentaose: 2’-FL: 2’-fucosyllactose; 3’-SL: 3’-sialyllactose; 6’-SL; 6’-sialyllactose; LA: Linoleic acid: ALA: α-Linolenic acid; DHA: Docosahexaenoic acid; LNT: lacto-N-tetraose LNT; LNnT: lacto-N-neotetraose; HIV: Human Immunodeficiency Virus; CS: caesarean section.
